# Dietary Supplementation of *Bacillus subtilis* as Probiotic Influenced the Growth Performance, Hematological Parameters, Immune Function, Antioxidant Status, and Digestive Enzyme Activity of Nile Tilapia Fingerlings (*Oreochromis niloticus*)

**DOI:** 10.3390/ani15091256

**Published:** 2025-04-29

**Authors:** Hairui Yu, Sadia Nazir, Farah Ijaz, Muhammad Umer Zahid, Maida Mushtaq, Muhammad Khan, Abdur Rahman, Muhammad Aziz Ur Rahman

**Affiliations:** 1 Weifang Key Laboratory of Coho Salmon Culturing Facility Engineering, Institute of Modern Facility Fisheries, College of Biology and Oceanography, Weifang University, Weifang 261061, China; abdurrehman@uvas.edu.pk; 2 Department of Fisheries & Aquaculture, University of Veterinary and Animal Sciences, Lahore 54000, Pakistan; sadianazir824@gmail.com; 3 College of Veterinary Medicine, Huazhong Agriculture University, Wuhan 430070, China; farahijaz0786@webmail.hzau.edu.cn; 4 Department of Animal Nutrition, University of Veterinary and Animal Sciences, Lahore 54000, Pakistan; drumerzahid@gmail.com (M.U.Z.); khanbwn011@gmail.com (M.K.); 5 Yunnan Animal Science and Veterinary Institute, Kunming 650000, China; maida.ch17@gmail.com; 6 Institute of Animal and Dairy Sciences, University of Agriculture, Faisalabad 38000, Pakistan

**Keywords:** *B. subtilis*, growth promotor, dietary supplementation, growth, health, immunity, Nile tilapia

## Abstract

This study evaluated the impact of *Bacillus subtilis* supplementation on growth, antioxidant status, digestive enzyme activities, hematological parameters, blood biochemistry, and immune responses in Nile tilapia (*Oreochromis niloticus*) fingerlings. A total of 180 fingerlings (mean weight: 5 ± 0.5 g) were randomly assigned to four groups: a control group and three probiotic-supplemented groups with 10^6^ (S-1), 10^8^ (S-2), and 10^10^ (S-3) CFU g^−1^ of *B. subtilis*, following a completely randomized design. Fish were fed twice daily for eight weeks. Results showed that the 10^10^ CFU g^−1^ group exhibited significantly higher weight gain (43 g), specific growth rate (2.5%/day), and better feed conversion ratio (0.7) compared to the control (30 g, 2.2%/day, and 1.4, respectively). Hematological parameters (WBC, RBC, hemoglobin) and immune responses (phagocytic activity, lysozyme) were significantly improved. Blood biochemistry showed decreased ALT/AST levels and increased ALP activity. Digestive enzymes (protease, lipase, amylase) and antioxidant activity (GPx, CAT, SOD) were enhanced, with reduced MDA levels. No significant differences were observed in survival rates or stress responses. These findings suggest that *B. subtilis* supplementation at 10^10^ CFU g^−1^ improves growth, immune function, and antioxidant status, making it a promising probiotic for Nile tilapia.

## Simple Summary

Probiotics are widely used in aquaculture to enhance growth, immunity, and overall fish health. This study found that *Bacillus subtilis* supplementation at 10^10^ CFU g^−1^ improved growth, feed efficiency, blood health, immune responses, and antioxidant status in Nile tilapia fingerlings over eight weeks. Fish in this group showed better weight gain, enzyme activity, and disease resistance. Survival rates remained unchanged. These results suggest *B. subtilis* as a beneficial probiotic for enhancing fish health and performance in aquaculture.

## 1. Introduction

Aquaculture is a crucial sector for global food security, supplying nearly 50% of the fish consumed worldwide, with an increasing trend due to the growing global population and declining wild fish stocks [1]. Intensive aquaculture practices, while enhancing production efficiency, often lead to challenges such as high stocking densities, competition for resources, and poor water quality, which consequently result in physiological stress, oxidative damage, suppressed immunity, and heightened susceptibility to diseases in fish [2,3]. The overuse of antibiotics to manage bacterial infections has further exacerbated concerns regarding antimicrobial resistance, environmental contamination, and bioaccumulation in aquatic organisms, prompting regulatory restrictions on their application in aquaculture [4,5]. As a result, there has been a significant shift toward finding safer, eco-friendly alternatives, with probiotics emerging as a promising approach to enhancing fish health, growth, and disease resistance [6,7].

Probiotics are live microorganisms that confer health benefits to the host when administered in adequate amounts, primarily by modulating gut microbiota, improving nutrient assimilation, and strengthening immune responses [8]. Among various probiotic candidates, Bacillus species have gained considerable attention due to their spore-forming ability, resilience to harsh environmental conditions, and diverse metabolic activities that contribute to improved fish health [9]. The administration of Bacillus spp. in aquaculture has been shown to enhance intestinal microbial balance, increase digestive enzyme activity, promote growth performance, and strengthen the host immune defenses of Nile tilapia [10,11]. Furthermore, Bacillus-based probiotics have demonstrated the ability to inhibit pathogenic bacteria through competitive exclusion and the production of antimicrobial compounds such as bacteriocins and lipopeptides [12,13]. However, the efficacy of probiotics is strain-dependent, and inappropriate probiotic strains may lead to unintended consequences, such as immune overstimulation, horizontal gene transfer, and the potential for systemic infections [14,15]. Therefore, an ideal probiotic should be derived from the host’s microbiota, possess strong colonization abilities, promote beneficial bacteria, and suppress opportunistic pathogens without adverse effects [16,17].

Among Bacillus species, Bacillus subtilis has been widely studied for its multifaceted benefits in aquaculture. It has been reported to enhance antioxidant activity, improve digestive efficiency, and modulate immune responses in various fish species [18,19]. Specifically, *B. subtilis* supplementation has been associated with increased feed conversion efficiency, higher growth rates, and improved survival in aquaculture species [20]. Moreover, studies indicate that *B. subtilis* enhances immune-related enzymatic activity, including lysozyme, peroxidase, superoxide dismutase (SOD), catalase (CAT), and immunoglobulin M (IgM), which are crucial for maintaining disease resistance in fish [21]. Researchers documented that the dietary supplementation of *B. subtilis* improved the growth performance, immune responses, and antioxidant status of Nile tilapia [22,23,24]. For example, Won et al. [22] reported the beneficial impact of combined *B. subtilis* and L. lactis at 10^8^ (CFU/g), while Hassaan et al. [23] found improved performance at a dosage of 25% of the diet. Despite the well-documented benefits of *B. subtilis* in aquaculture, there is still a dire need to explore the research on its application in Nile tilapia (*Oreochromis niloticus*), particularly optimization of dietary levels during the fingerling stage, as there is variation regarding dosage recommendations. Moreover, the drastic climate change in Pakistan makes it most vulnerable to environmental stressors and disease outbreaks for aquaculture species, as Pakistan has been facing significantly increased temperatures, altered precipitation patterns, deforestation, and water scarcity. In this context, Nile tilapia (*Oreochromis niloticus*) could be a promising species for aquaculture in Pakistan due to its adaptability to diverse environmental conditions, including varying water temperatures, low dissolved oxygen levels, and fluctuating water quality. Furthermore, the Nile tilapia’s relatively low environmental requirements and fast growth rate can contribute to food security and sustainable livelihoods and can enhance local economies while offering a viable solution to the increasing demand for fish protein in Pakistan. Keeping all this in view, the current study aimed to optimize the *B. subtilis* dosage in the practical diet of Nile tilapia fingerlings. The objectives of the study were to evaluate the impact of dietary *B. subtilis* supplementation at different inclusion levels on hematological and biochemical parameters, growth performance, antioxidant defense mechanisms, digestive enzyme activity, and the immune responses of Nile Tilapia.

## 2. Materials and Methods

### 2.1. Experimental Site, Approval, and Research Design

This experiment was carried out at the Fish Seed Rearing Unit, C block Ravi Campus Pattoki, and all experimental procedures were approved by the Animal Care and Use Committee of the University of Veterinary and Animal Sciences, Lahore, Pakistan (DR/163, 26-04-2021). In this experiment, 180 fingerlings (mean weight: 5 ± 0.5 g) were distributed across fifteen aquaria (n = 3 aquaria/treatment) and randomly assigned to four experimental treatments (n = 45 fingerlings/treatment) following a completely randomized design. The treatments consisted of a control group receiving a basal diet without supplementation and three groups fed probiotic-supplemented diets at concentrations of 10^6^ (S-1), 10^8^ (S-2), and 10^10^ (S-3) CFU g^−1^. These levels were selected based on the recommendation of the manufacturer. The probiotic, containing a single strain of Bacillus subtilis (BSN100) at a concentration of 1 × 10^9^ CFU, was procured from ECOSH company (Estonia). To achieve final concentrations of 1 × 10^10^, 1 × 10^8^, and 1 × 10^6^ CFU of *B. subtilis*, one gram of the probiotic (1 × 10^12^ CFU) was appropriately diluted. The required volume of the probiotic was calculated using the following equation and subsequently mixed with sterile distilled water:

Probiotic Volume = [Target CFU/Initial CFU] × Initial volume

The calculated volumes were then diluted with sterile distilled water. To confirm the CFU concentration in each prepared solution, a plate count assay was performed using nutrient agar plates (HiMedia Ltd., Lahore, Pakistan). The plates were incubated at 37 °C for 24 h, and the resulting colonies were enumerated using a digital colony counter (Model: AVI-35).

### 2.2. Dietary Ingredient Selection, Recipe Formulation, and Feed Preparation

**Figure 1:**
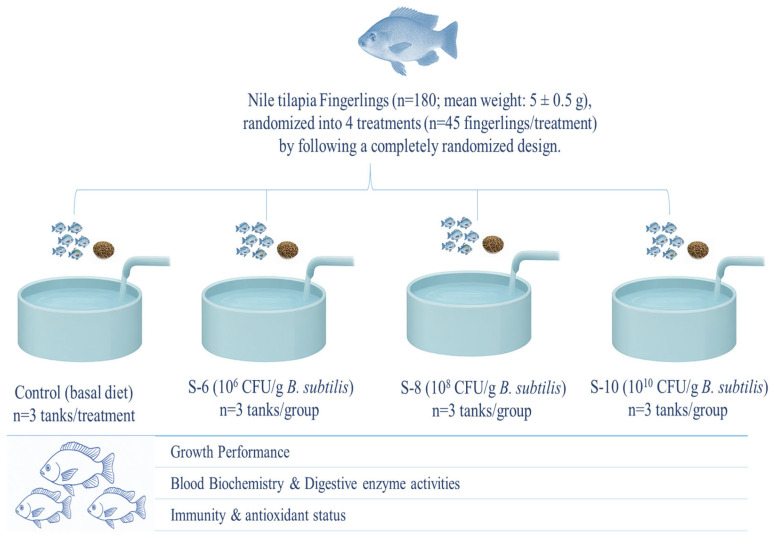
Experimental layout of the current study.

The dietary materials were purchased from the local market, and their composition and composite feed were assessed in accordance with Cunniff and Washington [24] guidelines. The ingredients were passed through a 0.05 mm sieve (KENWOOD, AT284, Solihull, UK), batched according to the inclusion levels of the basal diet ingredients, and supplemented with the designated Bacillus subtilis (1 × 10^6^, 1 × 10^8^, and 1 × 10^10^ CFU g^−1^) levels. Each batch was then thoroughly mixed using a KENWOOD AT283 mixer (KENWOOD, Solihull, UK), moistened with water to form a dough, and pelleted using a meat mincer (ANEX, AG 3060, Karachi, Pakistan). Bacillus subtilis was applied post-pelleting using an oil coating, ensuring minimal heat damage. The viability loss was less than 10%, as confirmed by CFU counts. The pelleted feeds were air-dried at room temperature and stored at 4 °C. To maintain probiotic viability, diets were freshly prepared weekly, and bacterial counts in the feed were assessed every three days using the plate count assay. The basal diet was formulated based on the nutrient requirements of Nile tilapia as per the Council [25]. If the concentration dropped below the desired level, freshly prepared probiotic feed was reapplied. Details of the dietary ingredient composition and chemical analysis are presented in Table 1. Moreover, the experimental design illustration is given in Figure 1.

### 2.3. Fish Husbandry Practices

At the start of the experiment, the fish were treated with a potassium permanganate solution (5 g/L) for 1–2 min to ensure biosecurity. The fish were divided into four groups and acclimatized in 120 L concrete tanks with a continuous water flow system. Tank water conditions were maintained at a pH of 7.2, a temperature of 27 °C, and a flow rate of 500 L/h. During the two-week acclimatization period, the fish were fed a basal diet (control) containing 36% crude protein at a feeding rate of 2% of their body weight per day. Health monitoring during acclimatization included visual assessment of skin and gill coloration, detection of external wounds, and observation of signs of illness. Only fish exhibiting normal skin and gill coloration, the absence of visible injuries or infections, and normal swimming behavior in flowing water were selected for the experimental trial. The fish selection criteria for uniformity in weight and health status were adapted and slightly modified from the method described by [26].

After acclimatization, the fingerlings were stocked in 15 aquaria (n = 15 fingerlings/100 L aquaria). Healthy fish were shifted into aquaria that were triple-stocked for each diet following the acclimatization period. During the experimental period, each aquarium’s dissolved oxygen level, temperature, and pH were maintained @ 5.8–7.3 mg/L, 24.9–28.7 °C, and 7.4–8.6, respectively. Fish were maintained under a 12 h light/12 h dark photoperiod throughout the experimental period. Moreover, each aquarium was subjected to a water change once a day. For 8 weeks, the experimental fish were fed twice a day at a feeding rate of 2% of their body weight.

### 2.4. Growth Performance Measurements

The mean weight of fish in all aquaria was recorded before the initiation of the feeding trial. Subsequently, the total weight of an equal number of fish from each aquarium (n = 15 fish) was measured weekly, and their averages were used to calculate the following growth performance. In addition, at the end of the trial, five fish from each aquarium (15 per dietary group) were randomly selected, and their individual body weights were recorded before blood collection to calculate the covariance. Growth performance parameters, including average weight gain (WG), specific growth rate (SGR), feed conversion ratio (FCR), and survival rate (SR), were then calculated using the formulas previously described by Mushtaq et al. [27].

Weight gain (g) = final body weight (g) − initial body weight (g)

SGR (%) = [(Final weight (g) − Initial weight (g)/days of growth trial] × 100

Feed conversion ratio = Feed intake (g)/weight gain (g)

Survival rate (%) = [Number of surviving fish/Initial number of fish] × 100

### 2.5. Sample Collection

At the end of the experiment, five randomly selected fish per aquarium were subjected to blood sampling via the caudal veins using 3 mL syringes and stored in EDTA vacutainers for hematological analysis. Additional blood samples were collected in Eppendorf tubes without anticoagulant and centrifuged at 3000 rpm for 15 min to collect serum, which was stored at −80 °C for biochemical analyses. Furthermore, three fish per aquarium (nine fish/group) were anesthetized with MS-22 following Mushtaq et al. [27], aseptically dissected, and their liver and gastrointestinal tracts (stomach, pyloric caeca, intestine) were collected for antioxidant and enzymatic analyses.

### 2.6. Hematological and Biochemical Analysis

Hematological parameters, including red blood cell (RBC) count, white blood cell (WBC) count, hematocrit (HCT), mean corpuscular volume (MCV), mean corpuscular hemoglobin (MCH), mean corpuscular hemoglobin concentration (MCHC), hemoglobin (HGB), and platelet (PLT) count, were analyzed using an autohematological analyzer (Celltac α, MEK-6550, Nihon Kohden Ltd., Shinjuku, Japan) at the General Laboratory, Department of Fisheries and Aquaculture, UVAS Ravi Campus, Pattoki. The serum was analyzed for AST (BioAST631), ALT (BioALT94), and ALP (BioALP2) using commercial kits (BioScien kits, Karachi, Pakistan) by following the protocols of Hossain et al. [28]. Additionally, serum samples were analyzed using an automatic biochemical analyzer (Hitachi 7600-110, Hitachi High-Technologies Corporation., Tokyo, Japan) to assess stress biomarkers, including cortisol and glucose levels. Immunological parameters, such as immunoglobulin concentration, total serum protein, lysozyme activity, and aminotransferase activity, were measured following the methodologies described by Ullah et al. [29]. Furthermore, fresh heparinized blood samples were analyzed for respiratory burst activity and phagocytic activity according to the protocols established by Solem et al. [30]. Briefly, isolated macrophages were incubated with NBT solution (0.1%) for 30 min at 25 °C. The reaction was stopped with methanol, and the cells were washed and air-dried. The reduced NBT (formazan) was then solubilized with 2 M KOH and DMSO, and absorbance was read at 620 nm using a microplate reader (Thermo Fisher, Waltham, MA, USA). For phagocytic analysis, isolated macrophages were incubated with heat-killed *Aeromonas hydrophila* stained with trypan blue at a ratio of 10:1 (bacteria/macrophage) for 1 h at 25 °C. The cells were then washed, fixed with methanol, and stained with Giemsa. At least 200 macrophages were observed under a microscope for the percentage of phagocytic activity.

### 2.7. Estimation of Digestive Enzyme Activities

The collected samples of gastrointestinal tissues were homogenized (1:10 *w*/*v*) in ice-cold phosphate buffer (0.1 M, pH 7.4), centrifuged (12,000× *g*, 20 min, 4 °C), and supernatants were used for enzyme assays. Protease activity was measured using casein as a substrate [31], with absorbance recorded at 280 nm, and one unit defined as the enzyme releasing 1 μmol of tyrosine/min. Lipase activity was determined using p-nitrophenyl palmitate [32], with absorbance recorded at 410 nm, and one unit defined as the enzyme releasing 1 μmol of p-nitrophenol/min. Amylase activity was assessed via the 3,5-dinitrosalicylic acid (DNS) method [33], with absorbance recorded at 540 nm, and one unit defined as the enzyme releasing 1 μmol of maltose/min. Total protein concentration was determined using the Bradford method for normalization.

### 2.8. Antioxidant Analysis

For antioxidant status, a 2 g liver sample was mixed with 6 mL of phosphate buffer (pH 7.4), filtered through Whatman filter paper no. 1, and centrifuged at 10,000× *g* for 15 min. The supernatant was separated, and all enzyme isolation procedures were performed at 4 °C. Catalase (CAT) activity was measured using the Ac [34] method by monitoring the decrease in H_2_O_2_ concentration at 240 nm with an Analytik Jena Specord 200 Plus UV/VIS spectrophotometer. Superoxide dismutase (SOD) activity was determined following Giannopolitis and Ries [35] by assessing its ability to prevent the photoreduction of nitroblue tetrazole (NBT). Glutathione peroxidase (GPx) activity was measured using the method of Civello et al. [36] by evaluating its ability to reduce H_2_O_2_ at 470 nm. Lipid peroxidation in muscles and liver was determined according to Mushtaq et al. [27], where samples were mixed with a KCl and Tris-maleate solution, followed by ascorbic acid, and heated with trichloroacetic acid. After cooling and centrifugation, thiobarbituric acid (TBA) and HCl were added, and TBA values were measured at 530 nm, expressed as µg malondialdehyde equivalents/mg tissue.

### 2.9. Statistical Analysis

The raw data were normalized using QQ plots (SPSS, Chicago, 18.0 version). The data were analyzed by one-way analysis of variance in SPSS software. The variation in the final body weight was used as a covariate for blood and other parameters. The means were compared using the Tukey test after adjusting the significance level set at *p* < 0.05.

## 3. Results

### 3.1. Growth Performance

The dietary supplementation of *B. subtilis* significantly influenced (*p* < 0.05) the growth parameters of Nile tilapia (Table 2), as these parameters showed a corresponding gradual increase with increasing supplementation of probiotics. Similarly, SGR was greater in the S-10-, S-6-, and S-8-supplemented groups compared to the control group (*p* < 0.05). Moreover, the feed conversion ratio was linearly improved with increasing levels of probiotic supplementation. However, the survival rate (%) was similar (*p* > 0.05) across the treatments.

### 3.2. Hematology

According to Table 3, the dietary supplementation of *Bacillus subtilis* influenced (*p* < 0.05) the hematological measurements of Nile tilapia. The values for WBC, RBC, HGB, HCT, and MCHC significantly increased (*p* < 0.05) as the level of *Bacillus subtilis* in the diets increased, with the highest values observed in the S-10-supplemented group. Additionally, MCV and MCH significantly decreased (*p* < 0.05) with increasing *Bacillus subtilis* supplementation in the diets compared to the control group. However, PLT values did not show any significant change (*p* > 0.05) with dietary supplementation.

### 3.3. Serum Biochemistry

In *Oreochromis niloticus*, dietary supplementation with *Bacillus subtilis* significantly (*p* < 0.05) reduced the activities of AST and ALT while significantly increasing the activities of ALP (Table 4). Fish fed S-6, S-8, and S-10 had significantly (*p* < 0.05) higher ALP activities than the control group. Conversely, AST and ALT activities showed a significant (*p* < 0.05) declining trend with increased *Bacillus subtilis* supplementation. The S-10 group exhibited the lowest AST and ALT activities, while the control group had the highest activities.

### 3.4. Immune Responses

The immunological and biochemical parameters of *Oreochromis niloticus* were significantly higher (*p* < 0.05) in the *Bacillus subtilis*-supplemented groups compared to the control group (Table 5). As *Bacillus subtilis* levels increased, IgM levels were significantly higher in the S-6-, S-8-, and S-10-supplemented groups compared to the control group (*p* < 0.05). The S-10-supplemented group exhibited the highest lysozyme activity. A similar trend (*p* < 0.05) was observed for respiratory burst (%) and phagocytic activity (%). The highest respiratory burst activity was observed in the S-10-supplemented group, while the control group had the lowest. Similarly, the highest phagocytic activity was observed in the supplemented group, with the lowest activity in the control group. Overall, the supplemented group exhibited the highest levels of IgM, lysozyme, respiratory burst, and phagocytic activity among the rest of the supplemented groups.

### 3.5. Digestive Enzyme Activities

The intestinal digestive enzyme activities (U/mg protein) of *Oreochromis niloticus* were significantly improved (*p* < 0.05) by the dietary addition of *Bacillus subtilis* (Table 6). Protease activity significantly increased (*p* < 0.05) with increasing *Bacillus subtilis* levels, with the S-6 group showing 12.8, the S-8 group showing 14.0, and the S-10-supplemented group showing 16.6, compared to the control group (10.8). A similar trend was observed for amylase and lipase activity (*p* < 0.05). The S-10-supplemented group had the highest lipase activity (4.96), significantly greater than the control group (1.93). Amylase activity was also highest (3.66) in the S-10 treatment, while the control group exhibited the lowest activity (1.73). Overall, the S-10 group exhibited the highest levels of lipase, amylase, and protease activity among the probiotic-fed groups.

### 3.6. Antioxidant Status

The diets supplemented with *Bacillus subtilis* significantly (*p* < 0.05) increased liver antioxidant enzyme activities, including SOD, CAT, and GPx in *Oreochromis niloticus* (Table 7). Fish fed S-6, S-8, and S-10 exhibited significantly higher (*p* < 0.05) CAT activities compared to the control group. Similarly, SOD activities were significantly higher (*p* < 0.05) in fish fed S-6, S-8, and S-10 compared to the control group. GPx activities were also highest (*p* < 0.05) in the S-10 group, while the control group had the lowest activities. Moreover, MDA concentrations were significantly lower (*p* < 0.05) in the *Bacillus subtilis*-supplemented groups compared to the control group.

### 3.7. Stress-Associated Circulating Biomarkers

Table 8 shows that the dietary supplementation of *Bacillus subtilis* did not influence the glucose and cortisol levels (*p* > 0.05). The blood glucose and cortisol levels were similar (*p* > 0.05) across the treatments.

## 4. Discussion

Most researchers have been actively investigating alternative, safer solutions to antibiotics for use in aquaculture, aiming to enhance fish production while minimizing negative impacts on the environment and human health. Among these alternatives, probiotics have emerged as natural bio-enhancers with strong potential to replace antibiotics due to their significant effects on fish health, growth, and disease resistance [37]. Probiotics benefit fish primarily by modulating gut microbiota, improving digestive efficiency, stimulating immune responses, and offering protection against pathogenic microorganisms. Among various probiotic candidates, Bacillus species are particularly noteworthy due to their growth-promoting, antimicrobial, and immunostimulatory properties. These bacteria are non-pathogenic and spore-forming, allowing them to survive harsh environmental conditions, which makes them ideal for inclusion in animal diets [38]. Their safety has been well documented, with no known pathogenic effects on host organisms. Several Bacillus species, including *B. subtilis*, *B. amyloliquefaciens*, *B. cereus*, *B. thuringiensis*, *B. pumilus*, *B. clausii*, *B. mojavensis*, *B. circulans*, and *B. licheniformis*, have demonstrated probiotic potential across various animal species, including fish [39,40,41]. These strains exert diverse beneficial effects, particularly on growth performance, immune function, and gut health. Despite its widespread use, limited research has focused specifically on *B. subtilis* as a probiotic for *Oreochromis niloticus* (Nile tilapia), especially during the vulnerable fingerling stage. Since fingerlings represent a critical growth phase, supplementation during this period may significantly influence early development, immune function, and disease resistance. Moreover, Nile tilapia possess a moderately developed stomach and relatively long intestine, consistent with their omnivorous feeding habits [10]. Therefore, this study aimed to evaluate the effects *of B. subtilis* supplementation at three different concentrations (10^6^, 10^8^, and 10^10^ CFU/g) on growth performance and health indicators in *O. niloticus* fingerlings over two months. The results demonstrated that *B. subtilis* supplementation significantly enhanced key growth performance parameters, including final body weight, average weight gain, specific growth rate, and feed conversion ratio, with the most pronounced effects observed at the highest concentration of 10^10^ CFU/g. These findings are consistent with previous studies reporting similar effects of *B. amyloliquefaciens* in *O. niloticus* [41], *B. licheniformis* in *O. mossambicus* [42], and *B. subtilis* in *Pangasius hypophthalmus* [43]. This supports the broader role of Bacillus species in enhancing fish growth and productivity. The improvement in growth performance can be attributed to the diverse bioactive mechanisms of *B. subtilis*, including the secretion of extracellular enzymes such as proteases, amylases, and lipases, which facilitate the breakdown of macronutrients and improve nutrient digestibility [11]. Additionally, the production of short-chain fatty acids, including acetate, propionate, and butyrate, contributes to gut health by supporting epithelial cell proliferation and serving as additional energy sources [2]. However, in some parameters, no statistically significant differences were observed between the control and treatment groups. This might be due to inter-individual variability, possible imbalances in gut microbiota, or enzyme activity, all of which could impact nutrient absorption and growth performance [44]. Moreover, the relatively short six-week duration may not have been sufficient to capture the long-term benefits of probiotic supplementation, indicating that extended trials are warranted.

In addition to growth, hemato-immunological indicators provided important insights into fish health. Hematological parameters such as HCT, RBC count, and WBC count are reliable indicators of the physiological and immune status of fish [45,46]. In the current study, fish supplemented with *B. subtilis*, especially at 10^10^ CFU/g, showed significantly elevated HCT, RBC, and WBC levels compared to controls. These findings align with prior research in *Cirrhinus mrigala* [47], *Oplegnathus fasciatus* [46], and *O. niloticus* [48,49], where probiotic supplementation enhanced hematological parameters. The increase in WBC count reflects immunostimulation and enhanced disease resistance [50], as probiotics such as Bacillus spp. are known to stimulate immune cell activity and modulate innate immune responses [50,51]. Liver and kidney biomarkers, including ALT, AST, and ALP, serve as key indicators of organ function and physiological well-being in fish [27]. In this study, *B. subtilis*-fed fish exhibited significantly lower ALT and AST levels, indicating reduced hepatic stress and improved liver function. This is consistent with earlier studies showing that probiotic supplementation, including *B. subtilis, B. licheniformis*, and *Enterococcus faecalis*, can decrease serum liver enzyme levels in fish [52,53]. Interestingly, ALP activity was higher in the probiotic groups, which may reflect increased macrophage enzyme activity and heightened immune readiness [54,55,56].

Key immune molecules, such as immunoglobulin M (IgM) and lysozymes, were also positively influenced by *B. subtilis* supplementation. IgM plays a pivotal role as the primary immunoglobulin in fish, while lysozyme contributes to bacterial cell wall degradation. In this study, fish receiving higher doses of *B. subtilis* displayed significantly increased serum IgM and lysozyme activity, particularly in the 10^10^ CFU/g group. This enhancement in immune function corroborates findings from *O. mossambicus* supplemented with *B. subtilis* [42] and reinforces the immunostimulatory capacity of the Bacillus species. Moreover, respiratory burst and phagocytic activities—key aspects of innate immunity—were significantly elevated in the probiotic-fed groups, echoing previous findings that suggest improved bactericidal capacity and immune defense following Bacillus supplementation [57,58,59,60,61]. Digestive capacity, another critical health indicator, was markedly improved through probiotic intervention in this study. Fish fed *B. subtilis* displayed significantly higher digestive enzyme activities, including protease, lipase, and amylase, enhancing nutrient breakdown and assimilation. These findings are in agreement with studies in *Litopenaeus vannamei* [62], *P. hypophthalmus* [63], *O. niloticus* [52], and *O. mossambicus* [42], highlighting the ability of Bacillus spp. to enhance digestive processes, thereby contributing to better feed utilization and overall performance. Probiotic supplementation also modulated antioxidant responses in this experiment. Fish fed *B. subtilis* showed higher activities of antioxidant enzymes such as SOD, GPx, and catalase, along with lower levels, indicating reduced oxidative stress and improved antioxidant defense. These results are consistent with earlier studies reporting similar protective effects of probiotics in various fish species [64,65,66]. The ability of probiotics to produce antioxidant metabolites, including glutathione and exopolysaccharides, further supports their role in mitigating oxidative damage [67]. Interestingly, no significant changes were observed in serum cortisol or glucose levels across the groups, suggesting that *B. subtilis* did not induce stress during the trial. These findings align with previous research showing no alterations in stress biomarkers in healthy, unstressed fish following probiotic supplementation [68]. These health-associated effects of *B. subtilis* can be attributed to its ability to secrete antimicrobial peptides such as subtilin, bacillomycin, surfactin, fengycin, and iturin, which inhibit pathogenic bacteria and help maintain gut microbial balance [7]. Additionally, the synthesis of bacteriocins enhances the competitive exclusion of harmful microbes [14]. *B. subtilis* also produces B-complex vitamins (e.g., B12, riboflavin, biotin), essential cofactors, and immunostimulatory metabolites that support metabolic and immune functions. Furthermore, quorum-sensing inhibitors produced by *B. subtilis* may reduce pathogen virulence, contributing to better intestinal health and immunity [16]. Collectively, these mechanisms underpin the observed improvements in nutrient utilization, immune defense, oxidative balance, and gut integrity, ultimately enhancing the growth performance and overall health status of *O. niloticus* fingerlings.

## 5. Conclusions

In conclusion, *Bacillus subtilis* supplementation, particularly at the 10^10^ CFU/g dose, improves the growth performance, immune response, and overall health of *O. niloticus* fingerlings. This study provides further evidence that probiotics, especially *Bacillus* species, have great potential as an antibiotic alternative in aquaculture, promoting fish health and growth without the negative side effects associated with antibiotics. However, further studies with longer trial durations and varied probiotic strains are necessary to confirm the long-term benefits of *B. subtilis* supplementation and its impact on fish health and production. Moreover, dietary supplementation might be more beneficial, particularly during disease outbreaks. Therefore, it is suggested that future studies that include pathogen challenge trials should be carried out.

## Figures and Tables

**Table 1:** Feed formulation and chemical composition on a dry basis.

Variables	Treatments ^1^			
	Control	S-6	S-8	S-10
Fish meal (%)	20.00	20.00	20.00	20.00
Soybean meal, 46 (%)	15.00	15.00	15.00	15.00
Sunflower meal (%)	15.00	15.00	15.00	15.00
Corn gluten 60 (%)	16.00	16.00	16.00	16.00
Wheat bran (%)	16.00	16.00	16.00	16.00
Rice bran (%)	16.00	16.00	16.00	16.00
Fish oil	1.00	1.00	1.00	1.00
Vitamin premix ^2^ (%)	0.50	0.50	0.50	0.50
Mineral premix ^3^ (%)	0.50	0.50	0.50	0.50
*B. subtilis* (CFUg^−1^)	0.00	10^6^	10^8^	10^10^
**Chemical composition**				
Dry contents (%)	89.25	88.93	89.03	89.31
Metabolizable energy (Mcal/kg)	2.97	2.97	2.97	2.97
Crude protein (%)	36.62	36.72	36.71	36.64
Crude fat (%)	9.22	9.07	9.10	9.13
Crude fiber (%)	8.32	8.27	8.29	8.31
Ash (%)	6.12	6.10	6.11	6.12

^1^ Treatments were a basal diet without any supplementation (control) and a basal diet supplemented with 6.0 (S-6), 8.0 (S-8), or 10.0 (S-10) g *Bacillus subtilis* per kg of the diet. ^2^ Each kg of vitamin premix contains vitamin A 15 M.I.U, vitamin D3 .I.U, nicotinic acid 25,000 mg, vitamin B1 5000 mg, vitamin E 6000IU, vitamin B2 6000 mg, vitamin K3 4000 mg, vitamin B6 4000 mg, folic acid 750 mg, vitamin B12 9000 mg, vitamin C 15,000 mg, and calcium pentothenate 10,000 mg. ^3^ Each kg of mineral mixture contains MgSO_4_·7H_2_O 153 mg, CoCl·6H_2_O 0.0816 mg, NaCl 51 mg, AlCl_3_·6H_2_O 0.255 mg, CuSo_4_·5H_2_O 210.67 mg, FeSo_4_·H_2_O 100.67 mg, MnSo_4_·5H_2_O 116.67 mg, ZnSO_4_·7H_2_O 121.33 mg, and cellulose 65 mg.

**Table 2:** Impact of *Bacillus subtilis* supplementation on growth performance and survivability of *Oreochromis niloticus*.

Parameters	Treatments ^1^				SEM ^2^	*p*-Value
	Control	S-6	S-8	S-10		
IBW (g/fish)	5.3	5.2	5.1	5.4	0.1577	0.1321
FBW (g/fish)	35.3 ^d^	40.6 ^c^	43.5 ^b^	48.4 ^a^	0.4041	<0.000
AWG (g/fish)	30.0 ^d^	35.4 ^c^	38.4 ^b^	43.0 ^a^	0.5773	<0.000
SGR (%)	2.2	2.3	2.4	2.5	0.0327	0.026
FCR	1.4 ^a^	1.3 ^b^	0.9 ^c^	0.7 ^d^	0.2309	0.053
SR (%)	100.0	100.0	100.0	100.0	-	-

^1^ Treatments were a basal diet without any supplementation (control) and a basal diet supplemented with 6.0 (S-6), 8.0 (S-8), or 10.0 (S-10) g *Bacillus subtilis* per kg of the diet. ^2^ SEM = standard error of means. ^a–d^ Superscripts within the same row were used from higher to lower order. The different superscripts mean significant difference between means at *p* < 0.05, while cells within the same row containing shared subscript have no statistically significant difference (*p* > 0.05). Initial body weight = IBW; final body weight = FBW; average weight gain = AWG; feed conversion ratio = FCR; specific growth rate = SGR; and survival rate = SR are acronyms.

**Table 3:** Hematological responses of *Oreochromis niloticus* supplemented with different levels of *Bacillus subtilis*.

Parameters	Treatments ^1^				SEM ^2^	*p*-Value
	Control	S-6	S-8	S-10		
WBC (10^6^/µL)	31.2 ^d^	31.47 ^b,c^	31.82 ^b,c^	32.00 ^a^	0.0219	0.053
RBC (10^6^/µL)	2.02 ^d^	2.35 ^b,c^	2.43 ^b,c^	2.59 ^a^	0.0372	0.072
HGB (g/dL)	7.2 ^d,c^	8.9 ^b,c,d^	9.5 ^a,b,c^	10.8 ^a,b,c^	0.4618	0.005
HCT (%)	27.31 ^d^	29.54 ^c^	34.15 ^b^	38.62 ^a^	0.6557	<0.000
MCV	119.66 ^a,b^	118.29 ^a,b^	109.45 ^c^	109.79 ^c^	0.2728	<0.001
MCH	47.51 ^a^	43.23 ^b^	40.12 ^c^	35.43 ^d^	0.4618	<0.001
MCHC (g/dL)	26.5 ^c,b^	27.8 ^b,c^	29.1 ^a^	30.7 ^a^	0.5196	0.005
PLT	1.61	1.69	1.76	1.79	0.2136	0.886

^1^ Treatments were basal diet without any supplementation (control) and a basal diet supplemented with 6.0 (S-6), 8.0 (S-8), or 10.0 (S-10) g *Bacillus subtilis* per kg of the diet. ^2^ SEM = standard error of means. ^a–d^ Superscripts within the same row were used from higher to lower order. The different superscripts mean significant difference between means at *p* < 0.05, while cells within the same row containing a shared subscript have no statistically significant difference (*p* > 0.05). White blood cells = WBC; red blood cells = RBC; Hemoglobin = HGB; hematocrit = HCT; mean corpuscular volume = MCV; mean corpuscular hemoglobin = MCH; mean corpuscular hemoglobin concentration = MCHC; and platelets = PLT are acronyms.

**Table 4:** Serum biochemistry of *Oreochromis niloticus* fed a diet supplemented with varying levels of *Bacillus subtilis*.

Parameters	Treatments ^1^				SEM ^2^	*p*-Value
	Control	S-6	S-8	S-10		
ALP (U/L)	80.15 ^d^	82.27 ^c^	84.91 ^b^	91.01 ^a^	1.1351	0.044
AST (U/L)	15.94 ^a^	13.29 ^b^	12.19 ^c^	9.67 ^d^	1.2061	0.034
ALT (U/L)	37.12 ^a^	32.61 ^b^	27.01 ^c^	24.02 ^d^	1.9680	0.015

^1^ Treatments were a basal diet without any supplementation (Control) and a basal diet supplemented with 6.0 (S-6), 8.0 (S-8), or 10.0 (S-10) g *Bacillus subtilis* per kg of the diet. ^2^ SEM = standard error of means. ^a–d^ Superscripts within the same row were used from higher to lower order. The different superscripts mean significant difference between means at *p* < 0.05, while cells within the same row containing a shared subscript have no statistically significant difference (*p* > 0.05). Alanine transaminase = ALT; aspartate transaminase = AST; and alkaline phosphatase = ALP are acronyms.

**Table 5:** Immunological responses of *Oreochromis niloticus* supplemented with different dosages of *Bacillus subtilis*.

Parameters	Treatments ^1^				SEM ^2^	*p*-Value
	Control	S-6	S-8	S-10		
IgM (mg/mL)	8.62 ^b,c,d^	9.80 ^b,c,d^	11.42 ^a,b,c^	12.53 ^a,b^	0.3605	0.002
LYZ (μg/mL)	8.73 ^d^	10.23 ^c,b^	11.52 ^a,b,c^	12.42 ^a,b^	0.6936	0.009
Respiratory burst (%)	0.41 ^d^	0.82 ^c^	1.34 ^b^	2.43 ^a^	0.1516	0.001
Phagocytic activity (%)	1.72 ^d^	2.41 ^b,c^	2.92 ^b,c^	3.84 ^a^	0.0886	0.028

^1^ Treatments were basal diet without any supplementation (control) and a basal diet supplemented with 6.0 (S-6), 8.0 (S-8), or 10.0 (S-10) g *Bacillus subtilis* per kg of the diet. ^2^ SEM = standard error of means. ^a–d^ Superscripts within the same row were used from higher to lower order. The different superscripts mean significant difference between means at *p* < 0.05, while cells within the same row containing a shared subscript have no statistically significant difference (*p* > 0.05). Immunoglobulin = IgM; and lysozyme = LYZ are acronyms.

**Table 6:** Influence of *Bacillus subtilis* supplementation on intestinal enzymes activities of *Oreochromis niloticus*.

Parameters	Treatments ^1^				SEM ^2^	*p*-Value
	Control	S-6	S-8	S-10		
Protease (U/mg protein)	10.8 ^d^	12.8 ^c^	14.00 ^b^	16.6 ^a^	0.2158	0.002
Lipase (U/mg protein)	1.93 ^d^	2.83 ^c^	3.76 ^b^	4.96 ^a^	0.1362	<0.000
Amylase (U/mg protein)	1.73 ^c,d^	2.56 ^b,c^	2.93 ^a,b^	3.66 ^a,b^	0.5844	0.022

^1^ Treatments were basal diet without any supplementation (control) and a basal diet supplemented with 6.0 (S-6), 8.0 (S-8), or 10.0 (S-10) g *Bacillus subtilis* per kg of the diet. ^2^ SEM = standard error of means. ^a–d^ Superscripts within the same row were used from higher to lower order. The different superscripts mean significant difference between means at *p* < 0.05, while cells within the same row containing a shared subscript have no statistically significant difference (*p* > 0.05).

**Table 7:** Changes in the liver antioxidant status of *Oreochromis niloticus* supplemented with varying concentrations of *Bacillus subtilis*.

Parameters	Treatments ^1^				SEM ^2^	*p*-Value
	Control	S-6	S-8	S-10		
SOD (U/mg)	4.57 ^d^	6.15 ^b,c^	6.28 ^b,c^	8.02 ^a^	0.558	0.038
CAT (U/mg)	69.83 ^d^	79.01 ^c^	82.08 ^b^	85.64 ^a^	1.241	0.010
GPH-x (μU/mg)	89.52 ^b,c^	92.27 ^b,c^	101.25 ^a^	103.32 ^a^	4.764	0.038
MDA (mg/g)	2.97 ^a^	2.72 ^b^	2.55 ^c^	2.11 ^d^	0.119	0.025

^1^ Treatments were basal diet without any supplementation (control) and a basal diet supplemented with 6.0 (S-6), 8.0 (S-8), or 10.0 (S-10) g *Bacillus subtilis* per kg of the diet. ^2^ SEM = standard error of means. ^a–d^ Superscripts within the same row were used from higher to lower order. The different superscripts mean significant difference between means at *p* < 0.05, while cells within the same row containing a shared subscript have no statistically significant difference (*p* > 0.05). Glutathione peroxidase = GPH-x, catalase = CAT, superoxide dismutase = SOD, and malondialdehyde = MDA are acronyms.

**Table 8:** Impact of *Bacillus subtilis* supplementation on stress biomarkers of *Oreochromis niloticus*.

Parameters	Treatments ^1^				SEM ^2^	*p*-Value
	Control	S-6	S-8	S-10		
Cortisol (ng/mL)	9.51	9.53	9.48	9.39	0.0581	0.293
Glucose (g/dL)	46.82	46.63	46.41	46.27	0.2318	0.315

^1^ Treatments were a basal diet without any supplementation (control) and a basal diet supplemented with 6.0 (S-6), 8.0 (S-8), or 10.0 (S-10) g *Bacillus subtilis* per kg of the diet. ^2^ SEM = standard error of means.

## Data Availability

The data supporting the findings of this study are available on request from the corresponding authors.
